# Tfcp2l1 as a central integrator of hypoxia, dedifferentiation, and tumor progression

**DOI:** 10.1186/s13046-025-03501-9

**Published:** 2025-08-14

**Authors:** Cynthia Clemente-González, Amancio Carnero

**Affiliations:** 1 Instituto de Biomedicina de Sevilla (IBIS), HUVR/CSIC/Universidad de Sevilla, Seville, Spain; 2 CIBER de Cancer (CIBERONC), Instituto de Salud Carlos III, Madrid, Spain

**Keywords:** TFCP2L1, Senescence, Hypoxia, Dedifferentiation, Tumorigenesis, Cancer

## Abstract

The gene *Tfcp2l1* has emerged as a central player linking key processes in cancer biology: hypoxia, immortalization, dedifferentiation, and tumor progression. Originally identified for its role in maintaining pluripotency in embryonic stem cells, *Tfcp2l1* has been found to reappear in various cancers, especially under conditions of low oxygen (hypoxia). Hypoxia, a common feature of solid tumors, triggers the reactivation of developmental genes like *Tfcp2l1*, enabling cancer cells to dedifferentiate and adopt stem cell-like properties. This dedifferentiation facilitates the immortalization of cells—allowing them to bypass senescence and continue proliferating. *Tfcp2l1* contributes to this process by regulating transcriptional networks that suppress differentiation and support self-renewal. Its expression correlates with poor prognosis in several cancers, highlighting its potential role in tumor aggressiveness and resistance to therapy. By acting at the intersection of cellular plasticity, stress adaptation, and oncogenic transformation, *Tfcp2l1* may serve as a molecular bridge linking early developmental programs with malignant behaviors. Understanding how *Tfcp2l1* integrates signals from hypoxic stress and drives dedifferentiation could uncover new therapeutic targets aimed at reversing or halting tumor progression.

**Supplementary Information:**

The online version contains supplementary material available at 10.1186/s13046-025-03501-9.

## Cancer

When we use the term cancer, we refer to a highly heterogeneous group of diseases, multifactorial in origin, characterized by abnormal and uncontrolled cell growth that results in the invasion of adjacent tissues and/or spread to other parts of the body. It can begin in virtually any tissue and is one of the leading causes of death globally [1, 2].

It is a complex process in which tissue homeostasis is altered. It can be defined as a group of cells with an accumulation of genetic, epigenetic, transcriptional, and/or post-translational alterations that give rise to distinctive aberrant cellular behaviors, known as “hallmarks of cancer.” These include the autonomous maintenance of proliferation, unlimited replicative potential (evasion of cellular senescence), evasion of growth suppression signals and apoptosis, and the capacity for angiogenesis, invasion, and metastasis [3, 4]. In cancer, there is not only high intertumoral heterogeneity, but also marked intratumoral heterogeneity derived from the new alterations that the cells acquire as they divide, giving rise to cells with different phenotypes and genotypes. The tumor microenvironment contributes to the development of this heterogeneity, as it determines access to oxygen and nutrients, pH conditions, topology, etc., and therefore generates a selective pressure that favors clonal evolution in the tumor [5].

Finally, the tumor is not only composed of transformed cells but also encompasses healthy auxiliary cells, such as fibroblasts, immune system cells, and endothelial cells that can provide support to the tumor and actively contribute to tumorigenesis [3]. Cooperation and competition between tumor cells, or between these and auxiliary stromal cells, as well as the specific conditions of the microenvironment, determine the bioavailability of growth factors, the production and release of released signaling molecules, and the dominant clones in each tumor region [6]. At the top of the proliferative and evolutionary hierarchy of these tumor ecosystems are tumor/cancer stem cells (CSCs) [5].

### Cancer stem cells

**Fig. 1:**
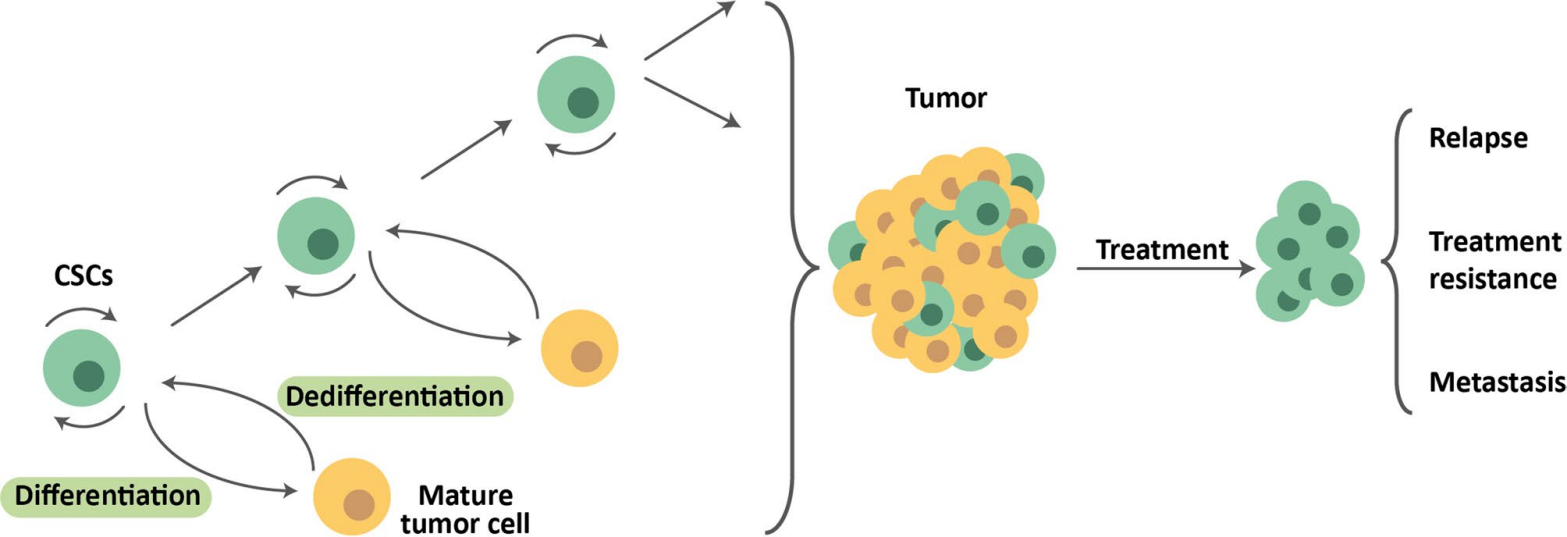
Tumor stem cell hierarchy. Tumor stem cells exhibit the capacity for self-renewal. Furthermore, they can divide, giving rise to more differentiated cells, which in turn can undergo dedifferentiation and give rise to CSCs again. The CSCs remaining after treatment may be responsible for recurrence, therapy resistance, and metastasis

CSCs were first identified in 1997, when it was determined that CD44 + CD24- cells from breast tumors were the only ones capable of replicating the disease once implanted in a mouse model [7]. Since then, CSCs have been identified and isolated from virtually all tumor types [8]. CSCs are capable of self-renewal and differentiation and generally share the typical characteristics of a normal stem cell, although the mechanisms that regulate these qualities are deregulated and therefore participate in tumor expansion [4]. Many of the signaling pathways active in these tumor stem cells are also common to those found in other pluripotent cells. Currently, CSCs are accepted as responsible for the initiation of tumorigenesis, tumor progression, migration, and metastasis [8–10]. Furthermore, these CSCs possess a series of characteristics and mechanisms that make them resistant to many of the therapies commonly used in cancer treatment, such as maintaining partial quiescence, greater efficiency in detecting and repairing DNA damage, protection against reactive oxygen species (ROS), increased anti-apoptotic pathways, and overexpression of proteins from the ATP-binding cassette (ABC) transporter family [11, 12]. For all these reasons, CSCs are also associated with relapse, and their elimination constitutes one of the main challenges when developing new antitumor therapies (Fig. 1).

The cells that make up the tumor display plasticity, transitioning into and out of the CSC state through dedifferentiation and differentiation, respectively (Fig. 1). The tumor microenvironment plays a fundamental role in this process of tumor dynamics. It has been observed that CSCs can influence nearby fibroblasts and convert them into what are known as cancer-associated fibroblasts (CAFs). Once this has occurred, the CAFs themselves contribute to CSC maintenance by promoting the WNT and NOTCH pathways [13, 14]. Other stromal cells, such as mesenchymal cells, are also capable of maintaining the CSC phenotype, in this case through the secretion of CXCL12, IL6, and IL8 and the activation of the NFκB pathway [15]. CSCs themselves modify the tumor microenvironment to replicate the conditions of stem cell niches, promote epithelial-mesenchymal transition (EMT) in nearby tumor cells, and alter the microenvironment of distant tissues by secreting factors such as VEGFA, LOX, TGFβ, and TNFα to promote metastasis [15].

The discovery of cellular reprogramming, as well as advances in the understanding of the pathways for acquiring and maintaining pluripotency, has had a significant impact on the development of theories about the origins of cancer. One of the proposed models is the stochastic theory, which argues that the accumulation of mutations in a somatic cell is capable of transforming it into any tumor cell able to reconstitute the tumor [16, 17]. On the other hand, the hierarchical model focuses on tumor stem cells, as they are the only ones with the capacity for pluripotency and self-renewal [18]. Although initially the hierarchical model seemed to be the most accepted, advances in the study of cellular de-differentiation processes have opened the possibility that differentiated tumor cells, through the activation of oncogenic transcriptional factors, acquire properties of self-renewal and pluripotency and are transformed into *de novo* CSCs [19, 20].

### Pluripotency and cell reprogramming

Stem cells are cells characterized by their capacity for self-renewal and differentiation into a multitude of functionally active cell types. Stem cells can be embryonic stem cells (ESCs), derived from the inner cell mass (ICM) of the embryo; or adult stem cells, responsible for the maintenance and regeneration of adult tissues. In stem cell biology, the term “potency” refers to the number of cell types into which a stem cell can differentiate. A pluripotent stem cell is, therefore, one capable of differentiating into cells derived from all three germ layers (endoderm, mesoderm, and ectoderm) [21].

When cultured in vitro under certain conditions, ESCs are able to maintain their pluripotent properties and their ability to generate lineages from all embryonic layers indefinitely. In these situations, the pluripotency pathways and self-renewal capacity remain active. Among the essential pathways for maintaining the pluripotent phenotype is the so-called “pluripotency core,” comprised of the transcription factors Oct3/4, Sox2, and Nanog [22]. The members of this pluripotency core act in a coordinated and interdependent manner. Oct3/4 regulates the expression of numerous pluripotency-related genes and often exerts this regulation by forming a dimer with Sox2. Some of the genes regulated by Oct3/4-Sox2 include Lefty1, Fgf4, and even the factors Oct3/4, Sox2, and Nanog themselves [22]. Oct3/4 expression requires extremely delicate regulation, as excessively high or low levels of the protein rapidly result in differentiation and loss of pluripotency [22]. Nanog levels, meanwhile, can vary among ESC populations, and some of its targets include Esrrb, Foxd3, Rest, and Rif1. In addition to the pluripotency core, other proteins play a key role in maintaining stem cell properties. These include SMAD1 and Signal Transducer and Activator of Transcription 3 (STAT3), and many of the coordinators of their activity, such as c-Myc, Esrrb, Klf4, and Tfcp2l1. The activity of all these proteins results in the activation of signaling pathways such as Leukemia Inhibitory Factor (LIF)/Stat3, Wnt/β-catenin, FGF/ERK, TGF/SMAD, and PKC [22].

Cells whose stemness is committed to a limited number of lineages can also acquire a pluripotent phenotype. This process, known as dedifferentiation or reprogramming, can be experimentally induced through various strategies (nuclear transfer, cell fusion, transduction with pluripotency factors) [23], although it can also occur spontaneously in the body under certain circumstances, causing diseases such as type 2 diabetes or cancer [24, 25]. Regardless of its origin, the reprogramming process involves a complete modification of the cell’s genetic and epigenetic landscape, including the expression of pluripotency-associated transcription factors and chromatin modifiers in perfectly orchestrated waves, chromatin remodeling, and transcriptome silencing associated with cell specification [26, 27].

One of the systems by which differentiated cells can be reprogrammed into induced pluripotent stem cells (iPSCs) is the transduction of the so-called “Yamanaka factors.” These are four transcription factors, Oct3/4, Sox2, Klf4, and c-Myc, whose exogenous overexpression triggers multiple signaling pathways that trigger cellular dedifferentiation until they return to the pluripotent stem cell state [28, 29]. Although this induced reprogramming can offer many advantages in personalized treatment and research, one of the main barriers to its routine application is the low efficiency of the procedure, sometimes achieving only 1% reprogramming efficiency. Additionally, reprogramming protocols are associated with a high oncogenic risk, mainly due to the activation of the c-Myc proto-oncogene and the use of viruses employed in the traditional protocol [30, 31]. Therefore, the study of the underlying mechanisms is of special interest.

### Cellular senescence

Cellular senescence is a state of proliferative arrest that differs from other cellular states in which the cell cycle is arrested (such as quiescence, cellular exhaustion, or terminal differentiation) by presenting a series of molecular, functional, and morphological alterations that constitute the senescent phenotype [32]. The evasion of this phenotype is a recurring feature in all tumors, constituting one of the Hallmarks of cancer [4].

Regarding molecular alterations associated with senescence, changes occur in the levels of proteins involved in cell cycle regulation, with high expression of cyclin-dependent kinase inhibitors (CKIs) of the INK4 and CIP/KIP families [33, 34]. In addition, levels of proteins with anti-apoptotic or survival functions increase, including the BCL-2 family, BCL-xL and BCL-w, PI3K, and p21CIP1 [35–37]. Senescent cells also undergo a high number of epigenetic changes at the level of both histone modifications and DNA methylation. The specific modifications depend on the specific factors that have induced senescence [38]. Other molecular alterations observed in senescent cells are those related to metabolism. Senescent cells show a high demand for energy and cellular components, resulting in an increase in glycolysis along with an increase in lipid and protein synthesis [39, 40]. All these genetic and metabolic changes lead to an increase in the synthesis and secretion of multiple molecules, giving rise to what is known as the senescence-associated secretory phenotype (SASP). This senescent secretome is composed of cytokines, chemokines, and proteases [35, 41]. The factors secreted by senescent cells vary depending on the cell type and tissue, although 55 factors common to all senescent cells have been identified, including VEGF, PDGF, HGF, IL1α, IL6, IL8, IL10, IL13, IL15, MMP3, MMP9, CXCL1, CXCL2, CXCL5, CXCL11, CXCL12, CCL2, and CCL20 [42].

At the functional level, elevated autophagic activity is observed in an attempt to compensate for the high energy demand and the presence of a large number of aberrant cellular organelles. Increased autophagic flux is associated with increased lysosomal activity, which in turn results in increased acid β-galactosidase activity. For this reason, increased acid β-galactosidase activity is commonly used as a marker of cellular senescence [36, 43]. Another affected cellular component is the mitochondria, which undergo alterations in membrane potential, structural modifications, and a greater tendency toward fusion than fission [44]. Morphological features of this phenotype include changes in the cytoplasm, which acquires a flattened and elongated appearance due to the overexpression of caveolin-1 [45]. Nuclear morphology is also affected, and senescent cells may be multinucleated or display irregular nuclei as a consequence of the loss of Lamin-B1 [46].

Senescence can be initiated in response to various internal or external stimuli, such as oncogenic signals (oncogene-induced senescence) [47], DNA damage and telomere shortening (replicative senescence), oxidative stress, ionizing radiation, or treatment with chemotherapy drugs (therapy-induced senescence) [36].

Although cellular senescence can be a pathological process and is sometimes associated with tissue aging [48], it is important to note that it is also required by the body to regulate processes such as structure formation during embryonic development, tumor cell elimination, tissue healing, and cellular reprogramming during tissue regeneration [49, 50].

#### Cell senescence and cancer

The role of senescence in cancer is complex, and multiple studies suggest that senescent cells may play a dual role in tumor progression [51]. The effect of senescence on cancer development depends on the intensity of the senescence trigger, the stage of the disease, and age [52]. Both antitumor and protumorigenic actions have been associated with the senescence response in virtually all aspects of tumor biology [53].

In the early stages, senescence acts as a tumor suppressor by reducing cell proliferation in response to elevated oncogene expression (OIS). Furthermore, senescent cells secrete a range of molecules that attract immune cells for elimination. Therefore, under these circumstances, preneoplastic cells not only exhibit limited proliferation but are also under intense immunosurveillance [54]. However, in more advanced tumor stages, the presence of a chronic inflammatory environment derived from an excess of senescent cells begins to play a pro-tumorigenic role. In vitro, cytokines produced by senescent fibroblasts stimulate the proliferation of malignant and pre-malignant epithelial cells and induce epithelial-mesenchymal transition signals [55]. In vivo, co-injection of senescent fibroblasts with tumor cells accelerates the appearance and progression of xenografts [56]. In patients treated with chemotherapy or radiotherapy, induced senescence in both the tumor and healthy tissues (treatment-induced senescence) has been linked to induction of pluripotency in tumor cells, relapse, metastasis, and a worse prognosis [57].

The SASP itself may have a different effect not only depending on the specific tumor stage but also on the cell of origin of the secreted factors. At the primary site, senescent tumor cells secrete factors that reinforce senescence arrest. At the same time, factors secreted by senescent fibroblasts and tumor cells can stimulate proliferation, angiogenesis, migration, and invasion. Furthermore, factors secreted by senescent stromal cells can promote the emergence of tumor stem cells. At the site of metastasis, SASP factors secreted by senescent osteoblasts create a pre-metastatic niche that facilitates tumor cell conquest and growth [58].

Finally, multiple studies have linked SASP-secreted molecules to the preparation of the pre-metastatic niche by inducing changes in the extracellular matrix, the recruitment and activation of stromal and immune cells with a pro-tumorigenic phenotype, metabolic reprogramming, and the restructuring of intercellular signaling pathways (15 and references therein). This effect is amplified by factors secreted into the environment by CSCs, especially those released in response to the hypoxic conditions commonly found in tumors.

## Hypoxia

“Normoxia” is defined as a situation in which the partial pressure of oxygen is equal to that normally found in the atmosphere, that is, around 21%. However, the availability of oxygen in different tissues of the body is more limited and is determined by factors such as blood flow and metabolic rate. Thus, well-irrigated organs such as the liver and kidneys can show oxygen levels of 4–14% [59], while in other organs such as the skin or brain, oxygenation is 0.5–4%. This situation of oxygenation below atmospheric levels, but normal under physiological conditions, is called “physioxia” [59, 60].

When access to oxygen is restricted beyond normal limits, we speak of a situation of “hypoxia.” Our body is prepared to respond to hypoxic situations through mechanisms that act at both the systemic and molecular levels. These mechanisms can be activated either in pathological situations (a decrease in available hemoglobin levels, vascular or respiratory failure, etc.) [61] or in non-pathological situations, as occurs after an increase in altitude and the associated decrease in atmospheric oxygen [62].

With respect to the molecular mechanisms of adaptation to hypoxia, the most relevant is mediated by hypoxia-inducible factors (HIFs). All cell types are capable of activating this pathway, in which the α subunit of HIF proteins is stabilized in hypoxia [61, 62]. This pathway is primarily regulated by post-translational modifications carried out by enzymes with prolyl hydroxylase domains (PHDs), which are sensitive to oxygen availability. Under normoxic conditions, sufficient oxygen is available for PHD proteins to hydroxylate one or two proline residues of HIFα subunits. There are three isoforms of the HIFα subunit (HIF1α, HIF2α, and HIF3α), all of which are susceptible to this hydroxylation. Removal of proline residues allows HIFα to undergo von Hippel-Lindau (VHL)-mediated ubiquitination, marking it for proteasome degradation. Under hypoxic conditions, prolines are not hydroxylated, and therefore, ubiquitination and subsequent degradation of the HIFα subunit do not occur. This allows it to dimerize with the HIF1β subunit, allowing both to translocate to the nucleus. There, the heterodimer binds to hypoxia-response elements in DNA and promotes gene expression that triggers various cellular responses and changes [62–64]. In situations where oxygen levels are even lower, another regulatory pathway mediated by the HIF1 inhibitory factor (HIF1) is activated. This protein is also sensitive to oxygen levels, and in this case, it is responsible for hydroxylating asparagine residues of HIFα under normoxic conditions. This hydroxylation prevents the transcriptional coactivators p300/CBP from interacting with HIF1 once in the nucleus, thereby affecting the activation of genes associated with the response to hypoxia. HIF1 can only regulate HIF1α in this way, while HIF2α is able to resist asparagine hydrolysis [62, 64, 65].

Some of the transcriptional targets of the HIFα-HIFβ heterodimer are common to all three HIFα isoforms, as is the case with GLUT1 and VEGF. Others are specifically regulated by heterodimers containing HIF1α, HIF2α, or HIF3α. This is associated with the functional and localization differences between the different isoforms. The HIF1α isoform is ubiquitous in the body and is responsible for the response to acute hypoxia, whereas HIF2α expression is restricted to certain cell types such as cardiomyocytes, epithelial cells, glial cells, and hepatocytes, and is active in chronic hypoxia. Specific targets of HIF1α include LDHA and PGK1, whereas HIF2α is capable of promoting the expression of EPO, MMP9, and OCT4 [66]. HIF3α, on the other hand, is expressed only in the brain, heart, kidneys, lungs, and thymus. Many of the HIF3α splice variants lack a transactivating domain, and it has been suggested that it could act as a negative regulator of HIF-associated transcription. One of the mechanisms through which it performs this function is through the sequestration of the HIF1α subunit after the formation of HIF1α-HIF3α, heterodimers incapable of activating the transcription of targets with hypoxia response elements [65, 67].

### Hypoxia and senescence evasion

The fact that HIF stabilization in hypoxia alters the expression of hundreds of proteins demonstrates the relevance and complexity of the HIF signaling pathway [61]. Furthermore, the genes transcriptionally regulated by HIF encode a wide variety of proteins with very different functions, including transcription factors, enzymes, transport proteins, ion channels, hormones, cytokines, and growth factors. This means that the response to hypoxia impacts a multitude of different essential processes, such as embryonic development, regeneration, cellular reprogramming, and stem cell biology [62]. Previous studies in cell culture have described how low oxygen concentrations can affect the proliferative lifespan of cells. For example, the proliferative lifespan of human fibroblasts under hypoxic conditions increases by 20% [68]. This effect extends to 80% in bovine fibroblast cultures [69] and to 500% in mouse embryonic fibroblasts (MEFs) [70].

It has traditionally been suggested that, in the case of MEFs, this effect could be directly related to accumulated oxidative damage, given that when cultured in a 3% pO_2_ ​​atmosphere, the damage would be less than when cultured under normoxic conditions [70, 71]. In this case, MEFs would be able to continue proliferating in hypoxia despite not showing alterations in the p19Arf/p53 pathway, two tumor suppressors whose gene disruption was considered essential for immortalization [72], and showing high expression of p16INK4a, a protein that at high levels usually activates entry into senescence [73]. Additionally, MEFs cultured in hypoxia, but with deficiencies in various proteins associated with DNA damage repair, suffered the usual proliferative arrest, although only if telomerase activity was not suppressed. All of this seemed to suggest that the accumulation of DNA damage under normoxic conditions could induce senescence in MEFs [74]. Another proposal for this effect is the possible existence of adaptive mutagenic responses to hypoxic conditions capable of extending proliferative lifespan. According to the theory of oxidative damage, the greater capability of human fibroblasts to repair this type of damage would explain the lesser effect of hypoxia on the proliferative lifespan of these cells [70, 74].

Another previously proposed explanation for oxidative damage is related to the metabolic changes resulting from culture in hypoxia. Under these conditions of low oxygen availability, glycolysis is favored over oxidative phosphorylation. This contributes to a reduction in oxidative damage and could thus facilitate cellular immortalization. An increase in the expression of the glycolytic enzyme phosphoglycerate mutase (PGM), with the corresponding increase in glycolytic flux, has been shown to prevent replicative senescence [75].

Although the traditionally accepted explanation was oxidative damage, in recent years it has been suggested that the evasion of senescence observed in hypoxia could be due to the activation of several signaling pathways under low-oxygen conditions. Specifically, the activation of pathways downstream of HIFα has been studied in depth, given that HIFα is the main regulator of the molecular response to hypoxia. Some of the suggested mechanisms have been p21Cip1 and c-Myc, both transcriptional targets of HIF1α and HIF2α [76, 77]; hTERT, which can be activated by HIF1α and plays a key role in the presence of ROS in mitochondria [78]; or p53 inhibition, which can occur both independently and dependently on HIF1α [79]. However, our laboratory has recently demonstrated that hypoxia is capable of extending the proliferative lifespan of MEFs through the activation of the CP2-like transcription factor 1 (Tfcp2l1). This protein is a transcriptional target of HIF1α and under low oxygen conditions, Tfcp2l1 expression levels increase [80].

The relationship between hypoxia and senescence is, however, complex and dual. It has been observed that, depending on the time and frequency of exposure to hypoxia, it can regulate processes that prevent senescence or act as a stressor capable of inducing it [81]. At the same time, senescence activates the SASP, a heterogeneous secretome composed of molecules capable of regulating proliferative lifespan in an autocrine and paracrine manner. Depending on the cell type that produces it, the composition of the SASP will be slightly different, and the proportions of each molecule will determine whether this regulation will act in favor or detriment of the evasion of senescence. Some highly relevant genes in the SASP that are transcriptionally induced by HIF1α include IL6, IL8, CXCR2, GROα, and PAI1 [82]. For example, IL6 plays a key role in inducing proliferation through the STAT3 pathway [83, 84], while secretion of CXCR2 and some of its ligands, including GROα, reinforce senescence activation by activating the p53 pathway [85].

### Hypoxia and maintenance of pluripotency

At the physiological level, hypoxia plays a key role in stem cells and their properties. During embryonic development, the embryo remains in a state of partial hypoxia throughout gestation, and evidence has been found of the relevance of hypoxia in maintaining the phenotype of ESCs [86]. Additionally, it has been described that stem cell niches, both somatic and embryonic, have a microenvironment in which oxygen levels are lower than those of the organs and tissues where they are located [87]. Furthermore, it has been shown that low oxygen levels contribute to the survival of hematopoietic stem cells and neural crest cells, as well as prevent ESC differentiation [88–90].

Culture under hypoxic conditions has been shown to promote the maintenance of pluripotency and self-renewal of both ESCs and other types of stem cells. Hypoxia also promotes the expression of pluripotency effectors and markers, including Oct3/4, Sox2, and Nanog, and consequently the proteins and signaling pathways downstream of these proteins. This effect can be HIFα-dependent or -independent [91].

The protein Tfcp2l1, a transcriptional target of HIF1α, binds to regions that regulate the expression of genes associated with proliferation or the stem cell phenotype, and recent work from our laboratory indicates that some of these targets could be Sox2 or Sox9 [80]. These genes had previously been shown to increase in expression in MEFs cultured in hypoxia, which was found to be associated with an increased proliferative capacity of these cells. Their expression was also elevated after overexpression at physiological levels of Tfcp2l1 in normoxia and reduced after its silencing in hypoxia [80]. Furthermore, other groups have previously reported that SOX2 levels are increased in response to hypoxia in adult stem cells, such as dental pulp cells, retinal progenitor cells, mesenchymal stem cells, and human ESCs (hESCs) in culture [92–94]. A similar finding is also found for SOX9 levels in response to hypoxia in bone marrow mesenchymal stromal cells and chondrocytes [95, 96], which may be one of the pathways by which hypoxia contributes to maintaining the pluripotent phenotype.

However, there is still controversy regarding the specific role of hypoxia in cell differentiation and dedifferentiation, probably due to the large number of factors that influence the process, such as the duration of exposure to hypoxia, the oxygen levels used, and the specific stage of maturation of the stem cells studied [91].

### Hypoxia and cellular reprograming

Hypoxia not only favors the maintenance of pluripotency in cells that already display this differentiation potential, but also facilitates the acquisition of the pluripotent phenotype by cells whose potency is limited. Numerous studies have shown that culture in hypoxia can increase the efficiency of reprogramming and even reduce the number of transcription factors required to initiate it. However, the specific mechanisms by which this phenomenon occurs have not yet been fully elucidated [97]. The theories proposed to date focus on the response triggered by HIF1α and HIF2α. Some of them focus on the metabolic changes undergone in hypoxia, specifically the transition to a metabolism based more on glycolysis and less on oxidative phosphorylation, as this phenomenon has also been described in iPSCs. However, the study of these processes is complex, as everything seems to indicate that each HIFα factor must be activated at a specific time point during reprogramming. For example, when HIF2α is stabilized toward the end of reprogramming, it inhibits the generation of iPSCs rather than inducing it [91, 98–100].

Initially, it was hypothesized that this facilitation of reprogramming observed in hypoxia could be due to the evasion of senescence, since senescence was previously thought to be merely an impediment to cellular reprogramming [101, 102]. This proposal was based on the fact that overexpression of Yamanaka factors triggered the activation of the p53, p16INK4a, p21CIP1, and DNA damage response (DDR) pathways. Additionally, the INK4a/ARF locus was remodeled and even appeared completely silenced in both mature iPSCs and ESCs. Furthermore, inhibition of all these senescence effectors resulted in increased reprogramming efficiency [101, 102].

However, other in vivo reprogramming studies have shown that senescence can enhance the efficiency of the process [103–105]. This may occur through the SASP since during the reprogramming process, not all cells undergo dedifferentiation, and, in some, overexpression of Yamanaka factors activates senescence pathways. Therefore, these cells initiate the secretion of SASP-associated factors, including IL-6. This interleukin has been shown to play a key role in reprogramming, as its release, along with the activation of its targets, increases reprogramming efficiency in non-senescent cells. This same process of increased reprogramming in vivo has also been observed in senescence induced by tissue damage or aging and appears to be dependent on p16INK4a but not on p53, p19Arf, or p21Cip1 [103–105]. However, as new theories are tested and new evidence is found, the relationship between hypoxia, senescence, and reprogramming appears to become more and more complex [106, 107]. We have described that one of the effectors responsible for increased reprogramming efficiency under hypoxic conditions is Tfcp2l1, a direct target of HIF1α. Furthermore, our results suggest that this phenomenon occurs as a consequence of transcriptional activation by Tfcp2l1 of genes such as Sox9, Sox2, Jarid2, and Ezh2, although the molecular mechanism has not yet been fully described [80].

### Hypoxia and cancer

One of the characteristics of tumors with rapid local progression is hypoxia. This is due to the fact that the blood vessels supplying the tumor are scarce and/or defective, so the amount of oxygen delivered is limited. Furthermore, not all tumor cells will have access to the same levels of oxygen, with the central and internal regions of the tumor being the most affected by hypoxia. Hypoxia is considered a marker of poor prognosis in cancer patients and promotes tumor progression. Although this has been described in depth, there is still controversy in the literature about whether, in these circumstances, hypoxia acts as a stressor that induces senescence in non-tumor cells or, on the contrary, contributes to tumorigenesis by facilitating immortalization prior to malignant transformation.

Hypoxia triggers cellular responses that result in increased tumor progression, activation of angiogenesis, metabolic reprogramming, promotion of metastasis, activation of mechanisms that generate resistance to therapy, and modulation of the immune response. For all these reasons, hypoxia correlates with increased disease aggressiveness and a worse prognosis, and it is extremely important to study its molecular effectors and their potential role as biomarkers or therapeutic targets [8, 108].

Previous studies [108–111] have shown that hypoxia can promote dedifferentiation into CSCs, as well as maintain the phenotype of CSCs already present in the tumor. Among others, some genes activated in response to hypoxia are the pluripotency-associated transcription factors OCT3/4, SOX2, NANOG, KLF4, and the NOTCH pathway. Epigenetic remodelers such as BMI1 and SIRT1 are also activated.

A close relationship has also been shown between hypoxia and CSC quiescence. Thus, cells located in hypoxic areas of the tumor display a dedifferentiated and quiescent phenotype, unlike what is observed in well-perfused areas [112]. One current proposal is that hypoxia promotes the dormant state of CSCs to increase their survival in the face of stress. For example, in prostate cancer, HIF1α has been shown to activate CXCR4 and NDRG1, normally regulated by n-MYC, to induce the dormant state [113, 114]. In glioblastoma, PP2A is activated by hypoxia, thus inducing cell cycle arrest at the G1/S point [115]. HIF1α also appears to be able to compensate for the pro-proliferative effect of c-MYC, causing cell cycle arrest and thus maintaining CSC identity [116].

The effect of hypoxia on CSCs can occur in two distinct ways: by limiting CSC differentiation or by promoting tumor cell dedifferentiation. In primary breast carcinoma, HIF1α increases the number of ALDH + cells and the population of CD44+/CD24 − cells [117]. Other effects of hypoxia on CSCs are not directly regulated by HIF, but rather by other hypoxia-associated proteins. Hypoxia increases the expression levels of the tumor suppressor VHL [118]. The protein MYBBP1A is a VHL ubiquitination target, and under hypoxic conditions, VHL marks MYBBP1A for ubiquitination. In renal cancer, decreased levels of MYBBP1A have been observed to increase c-MYB activity and, in response, induce the dedifferentiation of mature tumor cells into tumor stem cells [119, 120].

Both the HIF1α and HIF2α isoforms are important for the acquisition of stem cell properties in tumors under hypoxic conditions. However, tumors appear to prefer the action of the HIF2α isoform. Subpopulations with greater migration capacity in glioma lines show elevated levels of OCT3/4 and SOX2 induced by HIF2α [121]. Furthermore, in glioma models, the membrane marker CD44 releases its intracellular component, which interacts with HIF2α and increases the expression of pluripotency genes and their associated characteristics [122]. In certain leukemia models, NANOG and SOX2 facilitate MYC binding to the HIF2α promoter, and this maintains pluripotency by inhibiting p53 and ROS production [123]. Additionally, HIF2α activates LIF expression, with a high correlation between the two appearing in colorectal cancer patients [124].

**Fig. 2:**
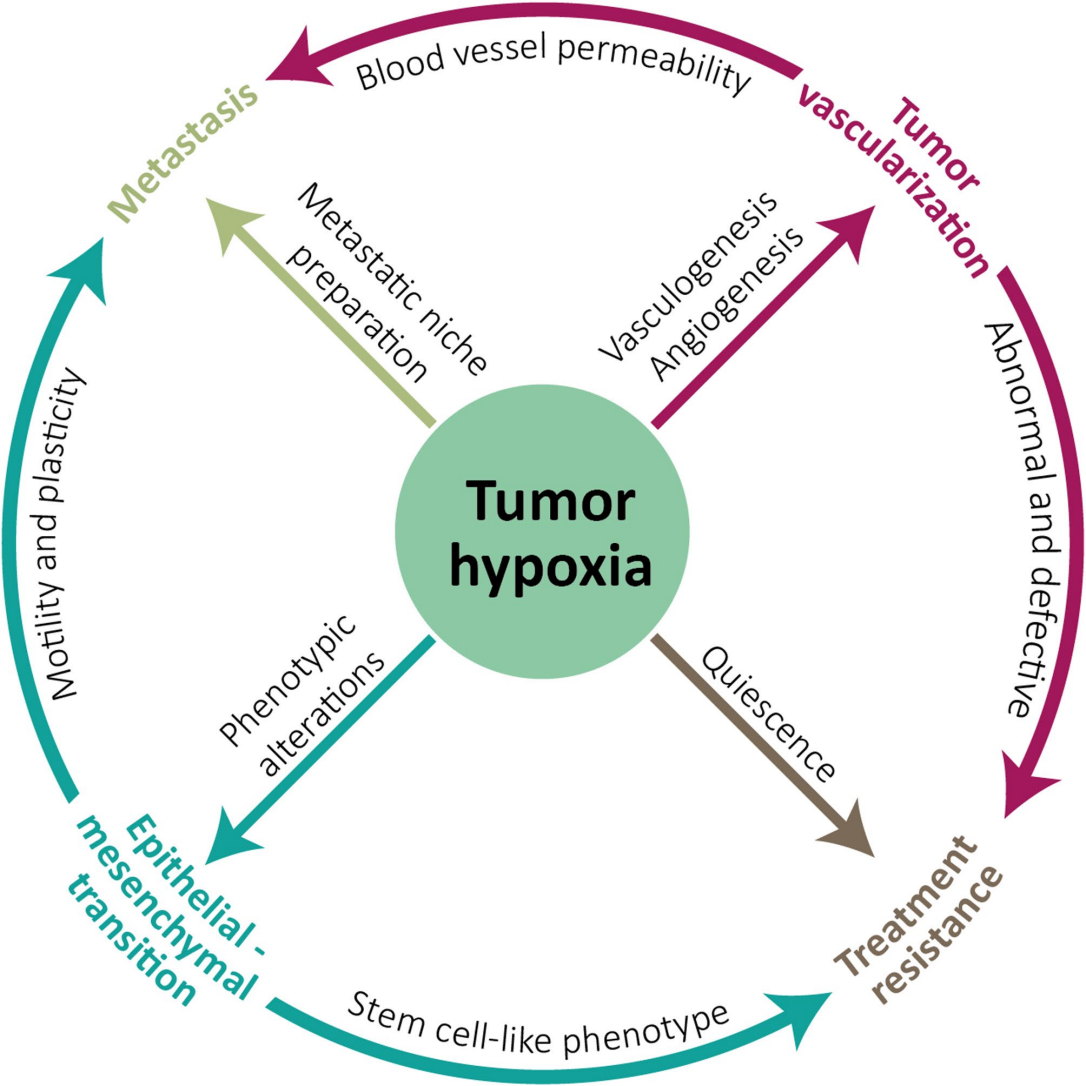
Effect of hypoxia on cancer. The interrelationship of different processes associated with tumor progression and affected by low oxygen concentrations is shown

Metastasis is also favored by hypoxia, as it increases the expression of EMT-related genes. The expression of these genes is also frequent in CSCs, and cells that undergo EMT often acquire stem cell capabilities, in addition to motility, dissemination and invasion capacity, and resistance to senescence, therapies, and elimination by the immune system [125]. Some of the genes activated by HIF1α are SNAI1, ZEB1, TWIST1, and TFC3 [126, 127]. For example, in breast cancer, hypoxia promotes EMT by activating ZEB1 and inhibiting MYB [128]. The expression of EMT genes is required for the appearance of circulating tumor cells (CTCs), cells that display CSC properties and can cause metastasis [129] (Fig. 2). Hypoxia also influences vesicle secretion, as low oxygen concentrations activate the expression of the exosome marker CD63 and the endosome-associated GTPase RAB22A. Under hypoxic conditions, these exosomes typically contain molecules such as VEGFR2, TNFα, β-catenin, AKT, and EGFR, which send signals that contribute to tumor progression, invasion, metastasis, angiogenesis, and immunosuppression [130, 131].

Even under normoxic conditions, tumors display greater use of the glycolytic pathway relative to oxidative phosphorylation, despite the former being less efficient. This is known as the “Warburg effect” and results in the high glucose consumption associated with tumors [132]. Under hypoxia, the Warburg effect represents an advantage that is further reinforced by the induction of glycolytic enzymes and the inhibition of mitochondrial metabolism through HIF. For example, HIF1α induces the transcription of PDK1, thereby preventing the transformation of pyruvate into acetyl-CoA and blocking its flow to the tricarboxylic acid (TCA) cycle [133]. It also activates the expression of LDHA, which diverts pyruvate into lactate instead of acetyl-CoA; and of PMK2, which participates in the last step of glycolysis and acts as a coactivator of HIF1α in promoting glycolysis and tumor growth [108]. Although transformed cells have been shown to express alternative isoforms of several of the glycolytic enzymes, HIF1α has also been shown to be able to promote their expression. These include ENO1 and HK2, which facilitate cell migration and inhibit apoptosis. HIF1α also induces the transcription of the GLUT glucose transporters [134, 135]. These transporters promote self-renewal and tumor initiation capacity and have been found to be overexpressed in CSCs of tumors such as glioblastoma, pancreatic cancer, and ovarian cancer [136].

The response to hypoxia also results in a series of epigenetic modifications that promote tumor progression. One of the enzymes whose activity is modulated by hypoxia is TET, which induces de novo methylation in EMT-associated genes such as INSIG1 [76, 137]. Under hypoxic conditions, the action of histone modifiers such as HDAC3 and WDR5, which mediate EMT in head and neck cancer and breast cancer cell lines, is also altered [138]. HIFα factors are also involved in the regulation of transcription of ALKBH5 and KNF127, which demethylate the 3’-UTR of KLF4 and NANOG messenger RNAs (mRNAs) and thus increase their expression in breast cancer [139].

## TFCP2L1

The TFCP2L1 protein (Transcription Factor Cellular Promoter 2 Like-1) is a transcription factor belonging to the Grainyhead-like (GRHL) subfamily of transcription factors [140]. The three members of this family, TFCP2, TFCP2L1, and UBP1, bind to the same DNA sequence, display high protein sequence homology, and are structurally similar. Although TFCP2L1 is the accepted terminology for HUGO, some authors also refer to it as CRTR-1, LBP-9, and, less frequently, LSF [141].

While the expression of TFCP2 and UBP1 is ubiquitous, TFCP2L1 is regulated spatiotemporally. It is primarily expressed in embryonic stem cells, the inner cell mass, and adult kidneys, although it may be present at lower levels in other tissues [142]. Depending on the target, TFCP2L1 can act as either an activator or a repressor of transcription [143].

In humans, TFCP2L1 cytogenetic band appears at position 2q14.2 [144], encoded by the minus strand, as shown in the Supplementary Fig. 1 gene maps obtained from Genecards (A), NCBI (B) and GRCh37-Ensemble (C).

In mice, TFCP2L1 cytogenetic band appears at chromosome 1 (position 1 E2.3; 1 52.14 cM), encoded by the plus strand. There are several regulatory elements near the *Tfcp2l1* coding region. Those elements include enhancers and active promoters, and using *Jaspar* tool the Hypoxia Response Element (HRE) sequence (5′-(A/G)CGTG-3′) was confirmed to be located in some of this promoters and enhancers. Specifically, one of them was located in a promoter downstream of *Tfcp2l1* coding sequence. The location of this HRE has not yet been described in detail.

**Fig. 3:**
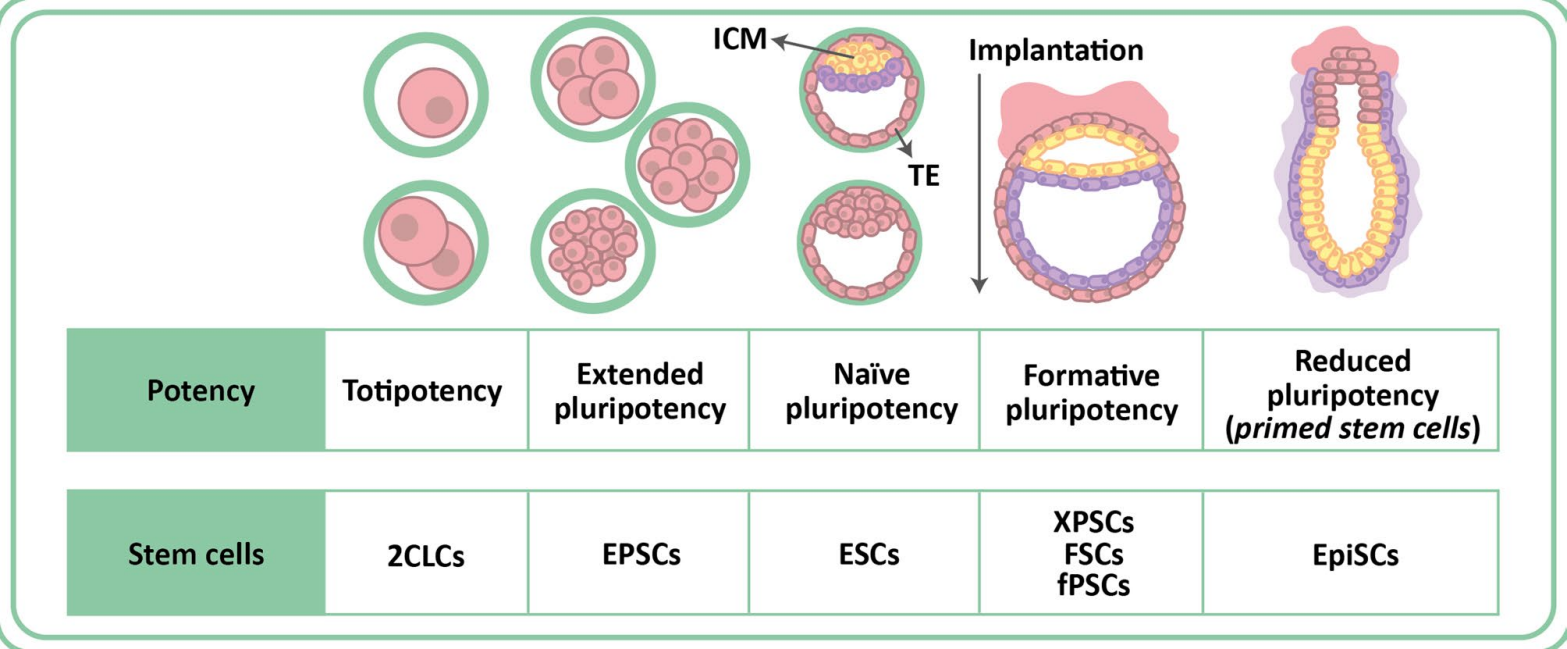
Classification of pluripotent stem cells based on their potency. Throughout embryonic development, pluripotent cells see their potency slightly restricted and are more predisposed to give rise to certain cell lineages. Totipotent two-cell-like cells (2CLCs) can give rise to both embryonic and extraembryonic tissues. The extended pluripotency of EPSCs allows them to display a certain potency both embryonic and extraembryonic. Naive pluripotent cells have not yet undergone any lineage specification, so their pluripotency is intact. On the other hand, cells with reduced potency (primed, EpiSCs) have already initiated a response to certain signals, and the fate of EpiSCs is considered to be partially specified. Cells with formative pluripotency represent an intermediate stage between naive and primed pluripotent cells. (Modified from Wu et al. 2022 [150]

Human TFCP2L1 is a protein composed of a CP2-like domain at the N-terminus and a SAM-like domain at the C-terminus, which are linked by a loop structure. The CP2 domain is involved in the binding of the transcription factor to its target sequence in DNA, while the SAM domain is involved in homo- and hetero-oligomerization with other proteins [142, 145]. TFCP2L1 forms hexadimers with itself, with each monomer weighing approximately 55 kDa, so the complete hexadimer appears to have a molecular weight of approximately 330 kDa [146]. The first function assigned to TFCP2L1 was its participation in embryonic development, a process during which it plays a determining role in the formation of the collecting tubules of the kidney [147] and the ducts of the salivary glands [148]. In both cases, the transcription factor acts by determining the pattern of cellular differentiation. It was later described that TFCP2L1, like other members of the GRHL subfamily, also participates in other processes, including reproduction (epigenetic reset and X chromosome reactivation), placental development, and the regulation of renal function and blood pressure (control of aquaporin-2 expression, regulation of electrolyte excretion in the kidney) [141]. In the last decade, TFCP2L1 has been recognized as one of the most important self-renewal factors in both human and mouse embryonic stem cells [146]. Tfcp2l1 levels are elevated in ICM stem cells, decreased in primed stem cells, and further reduced in differentiated cells [149, 150] (Fig. 3). Furthermore, the association of TFCP2 and UBP1 with several cancer types, coupled with the parallels between certain mechanisms of placental development and tumor growth, have led to the recent study of TFCP2L1 as a prognostic marker for cancer [141].

### Tfcp2l1 and maintenance of pluripotency

ESC self-renewal can be maintained through activation of the LIF/STAT3 pathway or through dual inhibition (2i) of glycogen synthase kinase 3 beta (GSK3) and mitogen-activated protein kinase (MEK) [151]. For example, the inhibitors CHIR99021 (CHIR) for GSK3 and PD0325901 (PD03) for MEK have been used. CHIR stabilizes β-catenin, allowing its translocation to the nucleus and thus the activation of the Wnt/β-catenin pathway. This signaling pathway suppresses the activity of Tfcp2l1, which is typically found to repress pluripotency networks [151]. PD03, for its part, blocks signaling downstream of the MEK/ERK pathway, although the specific mechanism of action is unknown. LIF can replace either PD03 or CHIR whenever present in the other [151].

The transcription factor Tfcp2l1 has been described as the factor at the convergence of both pathways, thus playing a critical role in maintaining pluripotent cell identity [151, 152]. The effect of both pathways in promoting self-renewal can be replicated by forced overexpression of TFCP2L1 [151], and the function of this protein cannot be compensated by overexpression of other LIF pathway targets. Furthermore, TFCP2L1 expression is necessary and sufficient to induce the molecular reprogramming of post-implantation epiblast stem cells toward naive pluripotency [152].

In the absence of a supporting cell layer, known as the Feeder Layer, LIF alone is unable to maintain ESC self-renewal; instead, the addition of 2i is required. Overexpression of Tfcp2l1, however, allows self-renewal in the absence of Feeder Layer with the sole addition of LIF [151]. The crucial factor provided by Feeder Layer appears to be bone morphogenic protein 4 (BMP4), which activates the Smad1/5/8-Smad4 signaling pathway [153]. Overexpression of Tfcp2l1 can maintain self-renewal in the absence of LIF/STAT3 signaling, whereas overexpression of other LIF/STAT3 pathway targets only partially recreates self-renewal, and silencing none of them is able to completely abolish self-renewal [151]. The contribution of Tfcp2l1 to maintaining pluripotency appears to be complex and multifactorial, with numerous mechanisms by which it could carry out this function having been described. On the one hand, TFCP2L1 forms a complex transcriptional network with the transcription factors OCT4, SOX2, and NANOG, also known as the “pluripotency core” [154]. TFCP2L1 directly regulates the transcription of Klf4 and Nanog [149, 155], in addition to physically interacting with OCT4, one of the central factors in the pluripotency transcription network [156]. Forced depletion of Klf4 also results in a blockage of Tfcp2l1’s participation in the induction of self-renewal and colony-forming capacity [149].

**Fig. 4:**
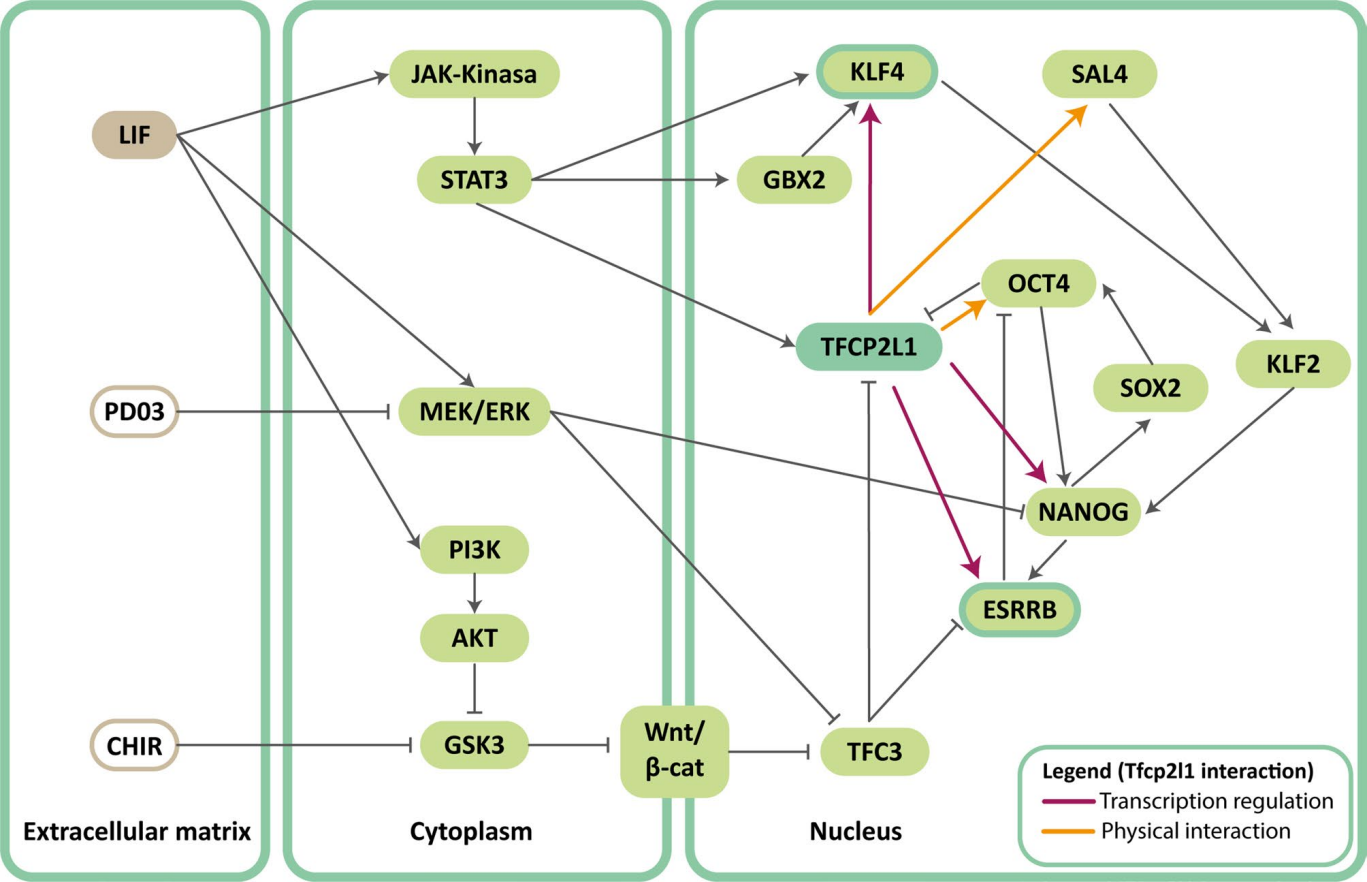
Role of TFCP2L1 in the circuit of essential pluripotency factors. Essential pathways in the maintenance of pluripotency in which the role of TFCP2L1 has been described are listed. (Modified from Dunn et al. 2014 [157]. Additional colors to the arrows represent the specific functional interaction between Tfcp2l1 and other transcription factors, reflecting the interactions described in the text

Another direct target of Tfcp2l1 is the estrogen-related receptor β (Esrrb), another transcription factor whose role in pluripotency has been previously described. Esrrb silencing eliminates the ability of Tfcp2l1 to induce LIF-independent self-renewal and reprogram epiblast stem cells to naive pluripotent cells. Both Tfcp2l1 and Esrrb are negatively regulated by the GSK3/TFC3 axis [149, 157] (Fig. 4).

Furthermore, it has been described that part of the effect of TFCP2L1 on the maintenance of pluripotency in mouse ESCs (mESCs) occurs through the suppression of genes specific to the endoderm (Sox17, Gata4, Gata6), mesoderm (T and Mixl1), and trophectoderm (Cdx2, Eomes, and Elf5) [158]. TFCP2L1 is also essential for the maintenance of hESCs, with the Wnt/β-catenin pathway being one of the pathways implicated [159].

The regulation of metabolism is also critical for the maintenance of pluripotency in ESCs, and Tfcp2l1 acts as a positive regulator of many metabolic pathways, primarily those related to lipid metabolism. Carnitine palmitotransferase 1a (Cpt1a), one of the rate-limiting enzymes of fatty acid oxidation, is a direct target of the transcription factor Tfcp2l1. The Tfcp2l1-Cpt1a axis protects ESCs from cell death under conditions of glutamine depletion or inhibition of glycolysis [160].

### Tfcp2l1 and celular reprogramming

Recently, it has been shown that Tfcp2l1 not only participates in the maintenance of pluripotency but also plays a crucial role in cellular reprogramming processes toward induced pluripotent cells. One of the underlying mechanisms described occurs through epigenetic remodeling. The DNA demethylase Tet2 is one of the most important epigenetic remodelers in the somatic reprogramming process, such that its overexpression increases reprogramming efficiency. Tfcp2l1 has been shown to recruit Tet2 to its target regions through direct interaction with the protein. This could be another mechanism by which increased Tfcp2l1 expression results in greater reprogramming efficiency [161].

Tfcp2l1 is also involved in the increased reprogramming efficiency observed under hypoxic conditions. Overexpression of Tfcp2l1 along with the four Yamanaka factors in MEFs undergoing reprogramming into iPSCs replicated the increased reprogramming efficiency observed when these cells were cultured in hypoxia. Additionally, silencing Tfcp2l1 using short hairpin RNA (shRNA) under hypoxic conditions reduced reprogramming efficiency to levels closer to those obtained in normoxia [80].

### Tfcp2l1 and senescence evasion

**Fig. 5:**
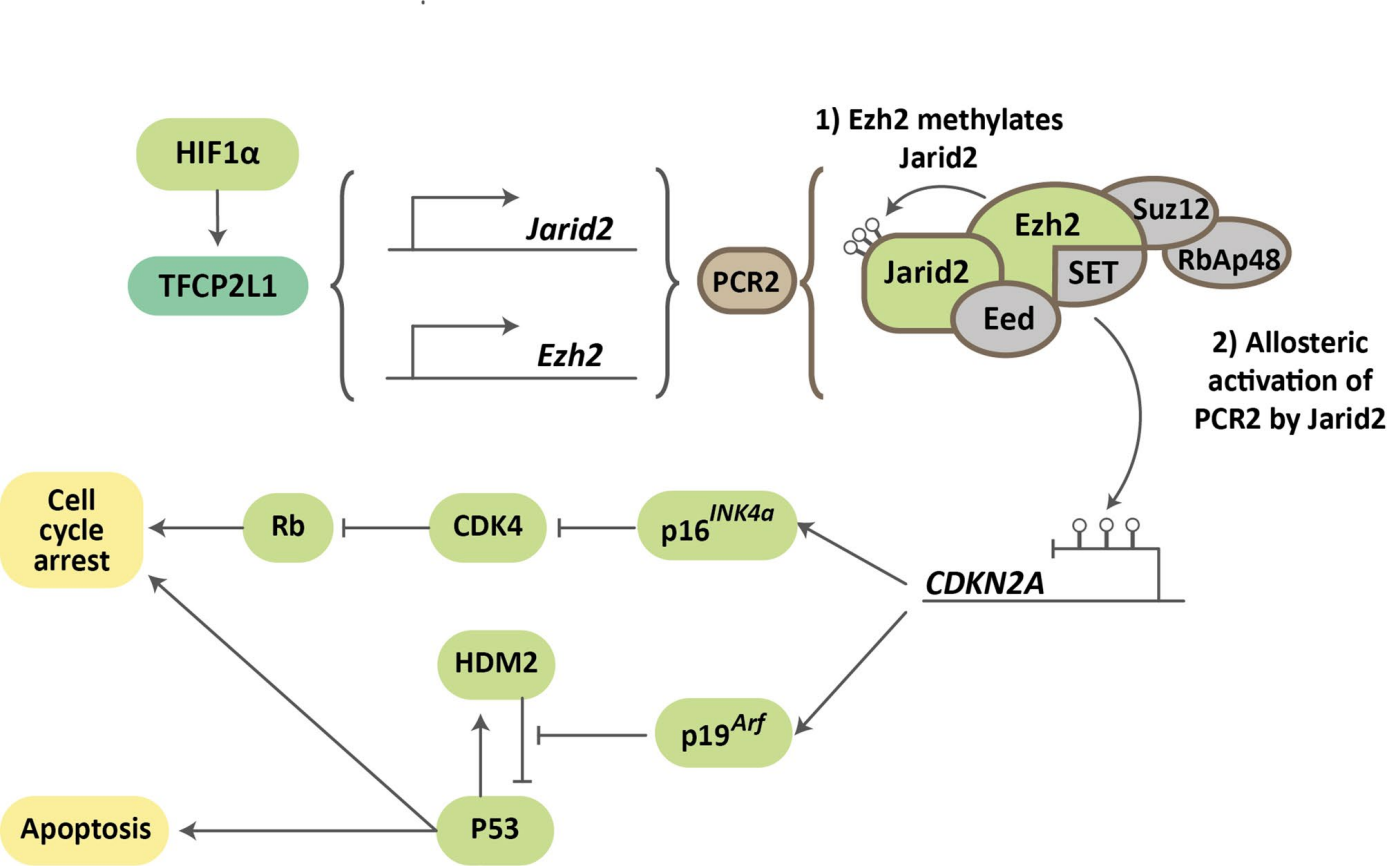
Role of Tfcp2l1 in senescence evasion. Proposed mechanism by which hypoxia, through the Tfcp2l1-Jarid2/Ezh2 pathway, could contribute to senescence evasion

As previously described, hypoxia is capable of promoting senescence evasion and inducing cellular immortalization. Recently, our laboratory demonstrated that the transcription factor Tfcp2l1 is transcriptionally regulated by HIF1α. Silencing Tfcp2l1 using shRNA halted MEF proliferation in hypoxia, prevented their immortalization, and resulted in the entry of these cells into senescence. Ectopic overexpression of Tfcp2l1, provided it was within levels similar to those induced by hypoxia exposure, replicated the results obtained by hypoxia: increased proliferative lifespan, immortalization, and evasion of senescence. Interestingly, when Tfcp2l1 overexpression was induced to supraphysiological levels, the effect was again an entry into senescence and loss of proliferative capacity [80]. These results seem to suggest that Tfcp2l1, like many transcription factors, especially those associated with pluripotency, performs its function within very specific expression ranges (Fig. 5).

Once expressed, Tfcp2l1 binds to regulatory regions of pluripotency-associated genes. However, our group showed that it also regulates the expression of genes associated with the control of senescence, such as Jarid2 and Ezh2, two members of the Polycomb Repressor 2 (PCR2) complex that are responsible for regulating the expression of the CDKN2A loci [80].

### Tfcp2l1 and cancer

There are numerous similarities between embryonic stem cells and human cancers, such as gene expression signatures, self-renewal capacity, and lack of differentiation. Some transcription factors involved in maintaining pluripotency also contribute to tumor development [154]. Moreover, members of the GRHL subfamily play different roles in multiple cancer types. For example, TFCP2 has been described as a pro-oncogenic factor in breast cancer, pancreatic cancer, and hepatocellular carcinoma, although it can also act as a tumor suppressor in other cancers such as melanoma. Furthermore, it promotes angiogenesis and participates in epithelial-mesenchymal transition processes [162].

Given its association with the pluripotent phenotype, TFCP2L1 could be related to the dedifferentiation and maintenance of CSCs, and it has been suggested that it could determine the effectiveness of chemotherapy [163]. However, studies conducted to date reveal that the role of TFCP2L1 in cancer is diverse, as in some tumor types it appears to act as a protective factor, while in others it is associated with a worse prognosis. This is consistent with the fact that factors associated with pluripotency are strongly dependent on their levels, range of action, and expression ratio relative to other genes involved in regulating the process. This dose-dependent effect appears to be well conserved in cancer. Added to this are the redundant roles that other related proteins can play, and the fact that cancer is not a single disease, but rather a collection of hundreds of individual diseases, each with its own genetic and epigenetic landscape [164].

In the case of clear cell renal cell carcinoma, TFCP2L1 expression is decreased, causing dysregulation of renal differentiation and promoting EMT [165]. The loss of TFCP2L1 in certain renal cells results in altered transcriptional regulation and malignant transformation into medullary renal carcinoma [166]. Levels of this transcription factor are also reduced in papillary thyroid cancer [167], and its expression negatively correlates with tumor stage and size, as well as with metastasis and extrathyroidal extension. The molecular mechanism by which TFCP2L1 acts as a protective agent in this case involves blocking the infiltration of pro-tumor immune cells by inactivating the NF-κB pathway [168]. Furthermore, CircHACE1 acts as a molecular sponge for microRNA (miR)-346, which consequently increases the expression of TFCP2L1 and results in an inhibition of cellular malignancy in thyroid cancer [169]. Increased expression of TFCP2L1 as a result of blocking CXCR2 activity in mouse models of melanoma resulted in decreased expression of genes involved in tumor growth and increased expression of genes associated with tumor suppression [170]. However, in hepatocellular carcinoma cells, reduced TFCP2L1 levels result in decreased proliferation, invasion, metastasis, and clone and sphere formation; whereas overexpression of the protein increases all of these properties. In this case, the effector pathway appears to be increased transcription of NANOG, which in turn results in greater activation of the JAK/STAT3 pathway [171]. Furthermore, studies suggest that elevated TFCP2L1 expression is a prognostic indicator of a high likelihood of lung metastasis in patients with basal-like breast cancer [172]. In bladder cancer, TFCP2L1 is phosphorylated by CDK1 and consequently represses ID2 transcription, which facilitates cell proliferation, tissue invasion and the development of pluripotent phenotype [173, 174]. Finally, high expression of Tfcp2l1 is considered a risk factor for survival in patients with glioma [175] and testicular cancer [176], and a marker of poor prognosis for melanoma and liver tumors [177].

## Conclusions

**Fig. 6:**
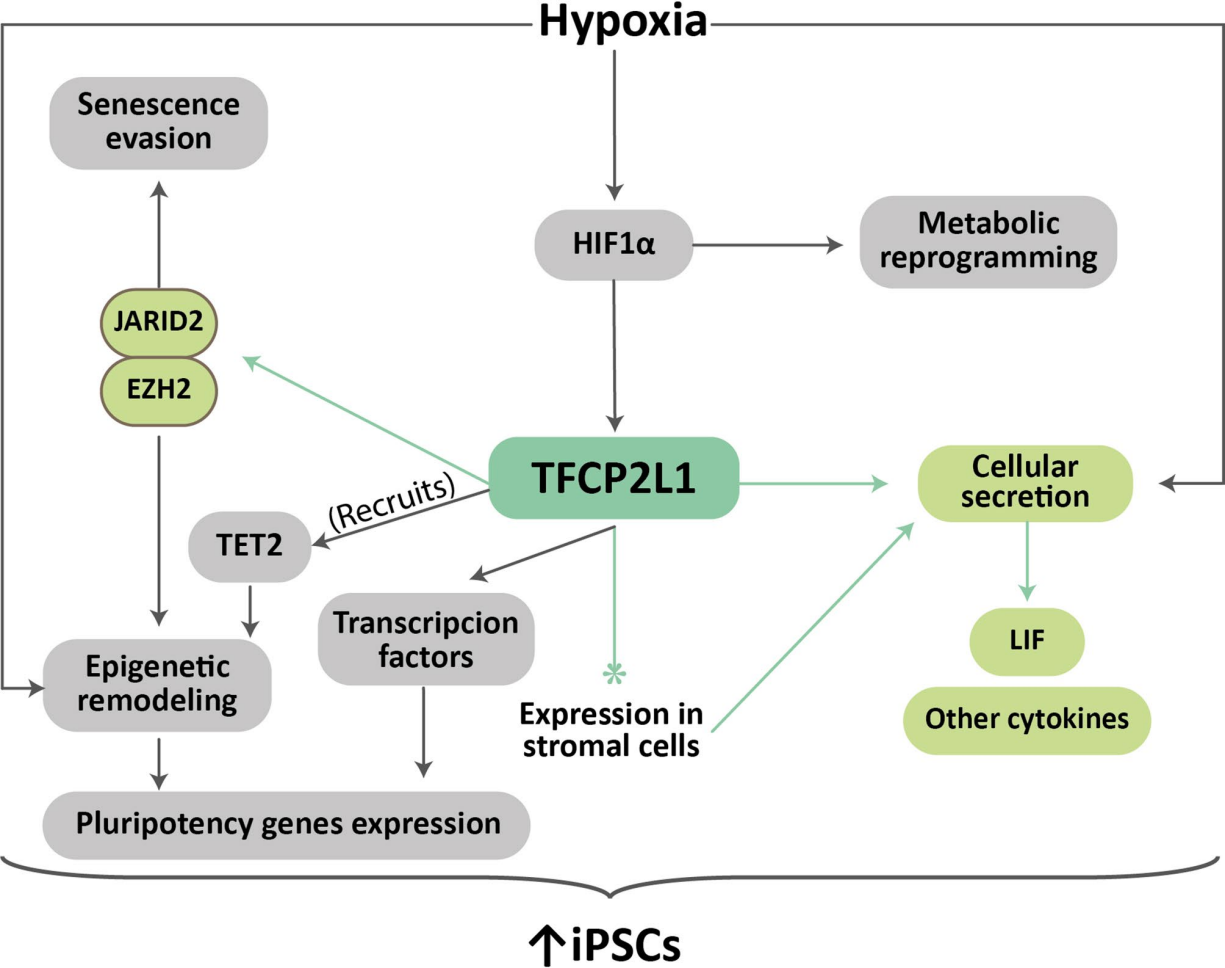
Summary of the role of TFCP2L1 in reprogramming efficiency. The mechanisms described by which hypoxia, through TFCP2L1, increases the efficiency of iPSC reprogramming are summarized

Cellular senescence is a complex cellular state characterized by a proliferative arrest along with a series of very characteristic phenotypic properties. This state can be induced by intrinsic and extrinsic mechanisms [35, 36] and plays a key role in numerous processes, both physiological (embryonic development, wound healing, tissue regeneration) and pathological (aging-related diseases such as Parkinson’s and Alzheimer’s diseases, atherosclerosis, osteoporosis, and osteoarthritis) [50]. It has previously been reported that culturing primary cells in hypoxia can prolong their proliferative lifespan and allow them to evade senescence processes [68–70]. The mechanisms underlying this fact are likely multiple and complex, which has led to several theories about its nature being proposed over the years, none of which have been able to fully explain this phenomenon. Recent studies have led to the proposal of a molecular mechanism by which primary culture of MEFs in hypoxia triggers Hif1α activation, elevating and maintaining the expression levels of the transcription factor Tfcp2l1 (Fig. 6). This, in turn, would bind to multiple enhancers and promoters distributed throughout the genome, many of them associated with genes involved in proliferation, pluripotency or immortalization [80]. And this Tfcp2l1-dependent transcription will be, at least partly, responsible for immortalization and dedifferentiation of mature tumor cells. Thus, the expression of this gene can contribute to worse prognosis in cancer, aggressiveness, metastasis, recidivas and therapy resistance in cancer. Therefore, the study of how to intervene effectively this gene could contribute to cancer therapies.

## Supplementary Information

Below is the link to the electronic supplementary material.

Supplementary Material 1

## Data Availability

No datasets were generated or analysed during the current study.

## Declarations

### AI statement

We used AI programs for correction and editing the English of this article.
