# Supplementary figures and images for: Tfcp2l1 as a central integrator of hypoxia, dedifferentiation, and tumor progression
Source: J Exp Clin Cancer Res. 2025 Aug 14;44:236. doi: 10.1186/s13046-025-03501-9 (PMC12351891; doi:10.1186/s13046-025-03501-9)

**Supplementary Figure 1:** gene maps obtained from Genecards (A), NCBI (B) and GRCh37-Ensemble (C).

A

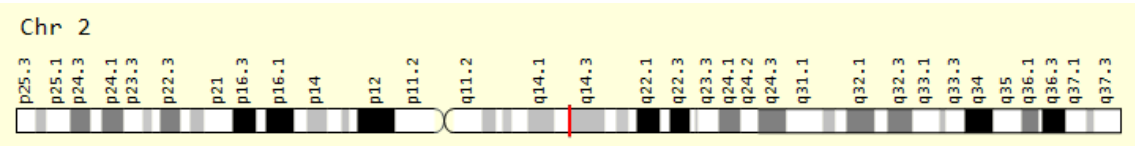

B

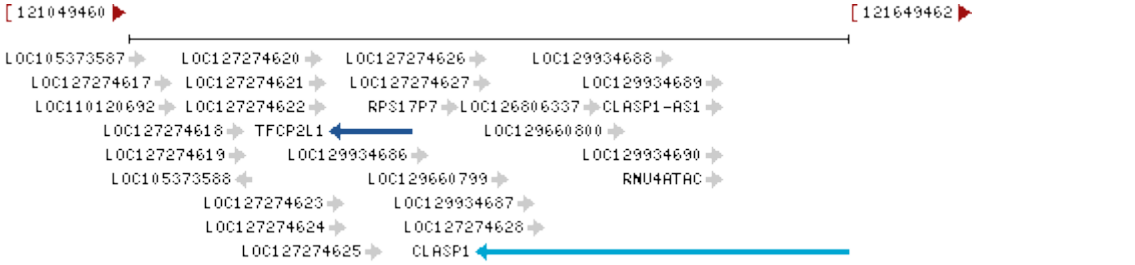

C

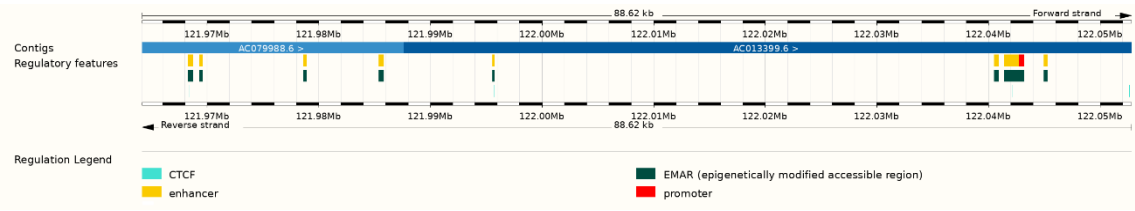

Supplement: Supplementary file 1 — Supplementary Material 1 [file 13046_2025_3501_MOESM1_ESM.pdf]
